# Epidemic risk perceptions in Italy and Sweden driven by authority responses to COVID-19

**DOI:** 10.1038/s41598-022-13218-w

**Published:** 2022-06-03

**Authors:** Elena Raffetti, Elena Mondino, Giuliano Di Baldassarre

**Affiliations:** 1 Centre of Natural Hazards and Disaster Science, Uppsala, Sweden; 2 Department of Earth Sciences, Uppsala University, Uppsala, Sweden; 3 Department of Public Health and Primary Care, University of Cambridge, Cambridge, UK; 4 Department of Global Public Health, Karolinska Institutet, Stockholm, Sweden

**Keywords:** Epidemiology, Public health, Psychology and behaviour

## Abstract

Understanding public risk perception is an essential step to develop effective measures reducing the spread of disease outbreaks. Here we compare epidemic risk perceptions during two different periods of the COVID-19 pandemic in Italy and Sweden. To this end, we analyzed the results of two nationwide surveys carried out in both countries in two periods characterized by different infection rates: August (N = 4154) and November 2020 (N = 4168). Seven domains of epidemic risk perception were considered: likelihood along with (individual and population) impact, preparedness, and knowledge. The role of the context and period was explored in stratified and formal interaction analyses. In both countries, we found an intensification in epidemic risk perception from August to November 2020. Being male, older and having a higher relative income were associated with a lower perception of the likelihood of epidemics, while excess mortality was marginally related to higher odds. Compared to Sweden, Italy had a higher increase in perception of likelihood and impact, and a concurrent decrease in preparedness and knowledge. The different authority response to the COVID-19 pandemic is associated with a different change over time in risk perception. Regional differences in terms of excess mortality only marginally explained differences in risk perception.

## Introduction

Untangling public risk perception is a pivotal point to support the development of policies promoting public health and reducing disaster risk. Individual experience, trust, knowledge, and coping appraisal, along with sociocultural indicators, influence how people think about risks^1–4^. A direct experience of a major crisis, such as the ongoing COVID-19 pandemic, strongly reshapes public risk perceptions. The availability heuristic can play a role in this process, as people tend to evaluate the importance, seriousness and probability of a threat based on how easily it comes to their mind^5^. Yet, while direct experience tends to increase risk perception, high levels of trust in authorities often result in a lower risk perception^1,6^. This is partially explained by the shift of responsibility from the individual to the trusted authority. Similarly, feeling knowledgeable about a risk may increase the individual’s coping appraisal, thus lowering their risk perception.

Along with a prompt response from authorities, public risk perception plays a crucial role to reduce the spread of disease outbreaks. Evidence from previous epidemics such as the Severe Acute Respiratory Syndrome (SARS, 2003) and H1N1 pandemic (2009) suggests that individuals comply with national recommendations proportional to their risk perception^7–9^. In particular, perceiving a high likelihood to be infected and a high risk of severe complications of the disease, along with trusting that government and authorities are recommending effective behavioral changes to control the spread, can shape how people adhere to national recommendations. Next to this, individuals may weigh the severity of COVID-19 infection effects to a different extent in relation to how scientists and media communicate the risk. The authorities’ underestimation of COVID-19 pandemic threat and the parallel with the seasonal flu at the beginning of the pandemic are emblematic of a risk communication strategy that facilitated the spreading of conspiracy theory using social media^10,11^.

**Figure 1:**
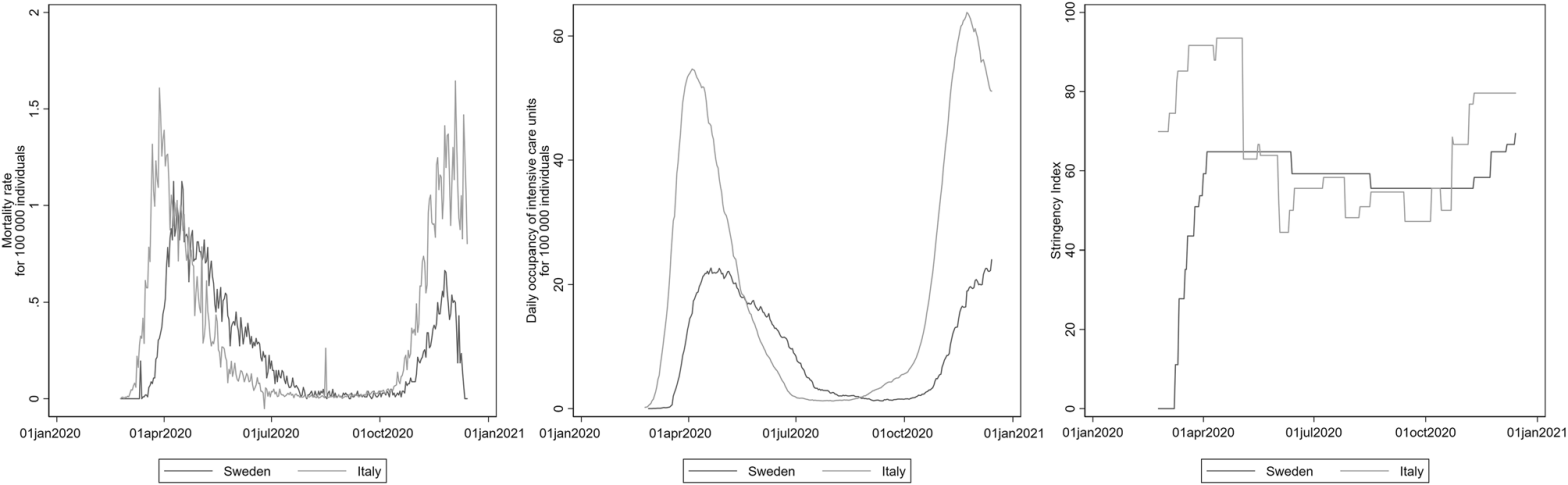
Mortality rate and daily occupancy of intensive care unit rate for COVID-19 per 100,000 individuals and the level of restrictive measures—the Stringency Index—in Italy and Sweden during 2020. The Stringency Index considers 9 domains (school closures, workplace closures, cancellation of public events, restrictions on public gatherings, closures of public transport, stay-at-home requirements, public information campaigns, restrictions on internal movements, and international travel controls).

Italy and Sweden were severely impacted during the first wave of the COVID-19 pandemic (spring 2020)^12^. The overall excess mortality was just below 30% in Italy and 20–25% in Sweden^12^. These dramatic figures can be partly attributed to the high proportion of people over 65 years in both Italy (23.0%) and Sweden (20.2%), which is substantially larger than the world average (9.1%)^13^, as well as initial outbreaks in densely populated areas, namely the Lombardy Region (418 inhabitants/km^2^) in Italy and the Stockholm Region (367 inhabitants/km^2^) in Sweden. The temporal evolution of COVID-19 deaths as well as daily occupancy of intensive care unit rates (ICU) in Italy and Sweden are presented in Fig. 1^14,15^. Both countries experienced a first epidemic peak in Spring 2020, which occurred in Italy a couple of weeks before Sweden, and the second peak in late Autumn 2020.

Although both Italy and Sweden have been highly affected by the first wave, the authority response and the distribution of the cases over time were different^12^. The world’s second COVID-19 outbreak after Wuhan occurred in the Lombardy region, in Italy. It strongly impacted the local health care system and resulted in a nationwide stay-at-home order for the general population from the 10th of March to the beginning of May^16^. This response prevented major COVID-19 outbreaks in Southern Italy, which was only marginally affected by the first wave, and led to a geographical pattern in the spread of infection. As a result, 71.0% of the estimated excess deaths during the first wave (i.e. between March and May 2020) occurred in three Northern regions, with a few provinces with excess mortality up to 800%^17^. Sweden was hit about two weeks later than Italy by the first COVID-19 wave (Fig. 1). In the second half of March, the Public Health Agency of Sweden recommended working from home and distance teaching (online) for high school and university students, along with a stay-at-home recommendation for risk groups (e.g. people older than 70 years) and those with respiratory symptoms^16^.

In this paper, we aim to unravel the role of authority responses on public perceptions of risk during the COVID-19 pandemic. To this end, we use observational data accrued in two countries (Italy and Sweden), which were severely affected by the COVID-19 pandemic, but with distinct authorities’ responses characterized by different levels of restrictions. First, we compare public perceptions of epidemic risks in Italy and Sweden during two different periods of the COVID-19 pandemic. Next, we consider multiple factors associated with the different domains of risk perception. Finally, we explore the role of national policies and excess mortality in influencing risk perception.

## Results

### Risk perception over time in Italy and Sweden

**Figure 2:**
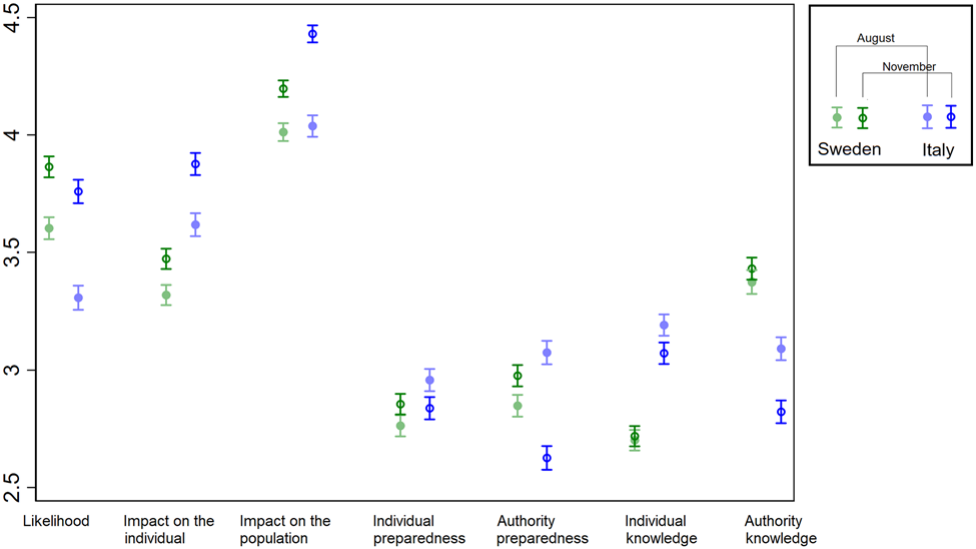
Means and 95% CIs for epidemic risk perception (likelihood, impact on the individual, impact on the population, individual preparedness, authority preparedness, individual knowledge, authority knowledge) by country and period.

We compared the risk perception of epidemics for seven domains between Italy and Sweden in two different periods of the COVID-19 pandemic (Fig. 2). Overall, the perception of how the epidemic impacted individuals, how individuals and authorities were prepared, and individual knowledge about epidemics were higher in Italy than in Sweden. Conversely, people in Italy perceived a lower likelihood of epidemics’ occurrence, and rated authorities’ knowledge on epidemics lower than people in Sweden. Generally, from August 2020 to November 2020, there was an overall increase in average risk perception, increasing the likelihood and impact of epidemics, along with decreasing perceptions of individual and authority preparedness and knowledge.

A formal interaction analysis supported a greater change in the magnitude of the associations in Italy compared to Sweden. Overall, a higher increase in perceived likelihood and impact, and a concurrent decrease in perceived preparedness and knowledge were observed (Table 1).

**Table 1:** Country (Italy vs. Sweden) effect modification on the association between period and pandemic risk perception.

Period	Sweden ORs (95% CIs)	Italy ORs (95% CIs)	ORs (95% CIs) for country within strata of period	Effect modification on additive scale RERI (95% CIs)	Effect modification on multiplicative scale ORs (95% CIs)
**Likelihood**					
August	1	0.63 (0.57,0.71)	0.63 (0.57,0.71)	0.15 (− 0.04,0.33)	1.36 (1.16,1.59)
November	1.55 (1.39,1.73)	1.33 (1.19,1.49)	0.86 (0.77,0.96)		
**Impact on the individual**					
August	1	1.79 (1.60,2.01)	1.79 (1.60,2.01)	0.75 (0.45,1.05)	1.23 (1.04,1.44)
November	1.29 (1.16,1.45)	2.84 (2.52,3.19)	2.17 (1.93,2.43)		
**Impact on the population**					
August	1	1.17 (1.05,1.31)	1.17 (1.05,1.31)	1.08 (0.79,1.36)	1.59 (1.35,1.87)
November	1.45 (1.30,1.62)	2.70 (2.40,3.04)	1.88 (1.67,2.12)		
**Individual preparedness**					
August	1	1.43 (1.28,1.60)	1.43 (1.28,1.60)	− 0.45 (− 0.66,− 0.24)	0.69 (0.59,0.80)
November	1.18 (1.06,1.32)	1.16 (1.03,1.30)	0.98 (0.88,1.10)		
**Authority preparedness**					
August	1	1.49 (1.33,1.66)	1.49 (1.33,1.66)	− 1.02 (− 1.25,− 0.80)	0.38 (0.32,0.45)
November	1.24 (1.11,1.38)	0.70 (0.63,0.78)	0.57 (0.51,0.63)		
**Individual knowledge**					
August	1	2.43 (2.17,2.72)	2.43 (2.17,2.72	− 0.52 (− 0.81,− 0.24)	0.77 (0.66,0.90)
November	1.05 (0.94,1.17)	1.95 (1.74,2.18)	1.86 (1.66,2.08)		
**Authority knowledge**					
August	1	0.62 (0.56,0.70)	0.62 (0.56,0.70)	− 0.30 (− 0.44,− 0.15)	0.60 (0.51,0.70)
November	1.08 (0.97,1.20)	0.40 (0.36,0.45)	0.37 (0.33,0.41)		

### Individual factors associated with risk perception

We found that the main factors related to risk perception were: (i) direct experience with epidemics and (ii) sociocultural factors such as gender, age, and relative income with a substantial stable association across countries and over time (Table 2). Here, we describe the association pattern.

**Table 2:** Factors associated with epidemic risk perception by country and period.

	Sweden		Italy	
	August	November	August	November
	OR (95% CI)	OR (95% CI)	OR (95% CI)	OR (95% CI)
**Likelihood**				
Males	0.58 (0.49,0.68)	0.54 (0.45,0.65)	0.86 (0.70,1.06)	0.71 (0.59,0.85)
Age 50–69 years	0.67 (0.55,0.80)	0.53 (0.43,0.66)	0.97 (0.78,1.21)	0.62 (0.51,0.75)
≥ 70 years	0.50 (0.37,0.68)	0.46 (0.34,0.61)	0.83 (0.56,1.22)	0.59 (0.43,0.80)
Employment	1.27 (1.00,1.62)	1.13 (0.90,1.42)	1.00 (0.80,1.25)	1.21 (0.99,1.47)
Relative income	0.96 (0.89,1.04)	0.91 (0.84,1.00)	0.89 (0.81,0.98)	0.99 (0.91,1.07)
University education	1.20 (1.01,1.43)	1.15 (0.95,1.38)	1.13 (0.91,1.41)	1.25 (1.03,1.51)
Experience of epidemics	3.16 (2.59,3.85)	3.51 (2.81,4.37)	3.16 (2.52,3.96)	2.49 (2.08,2.99)
**Impact on the individual**				
Males	0.80 (0.68,0.95)	0.58 (0.48,0.69)	0.69 (0.56,0.85)	0.71 (0.59,0.85)
Age 50–69 years	1.50 (1.24,1.81)	1.80 (1.45,2.23)	1.25 (1.01,1.56)	1.32 (1.09,1.61)
≥ 70 years	1.49 (1.08,2.07)	3.50 (2.59,4.72)	0.93 (0.64,1.36)	1.72 (1.27,2.32)
Employment	0.76 (0.59,0.97)	1.04 (0.83,1.32)	0.90 (0.72,1.13)	1.03 (0.85,1.25)
Relative income	0.87 (0.80,0.94)	0.89 (0.82,0.98)	0.83 (0.75,0.91)	0.96 (0.89,1.04)
University education	1.07 (0.90,1.28)	1.31 (1.08,1.58)	1.21 (0.97,1.51)	1.03 (0.85,1.24)
Experience of epidemics	1.45 (1.18,1.78)	1.27 (1.02,1.58)	1.22 (0.99,1.52)	1.52 (1.27,1.82)
**Impact on the population**				
Males	0.57 (0.49,0.68)	0.50 (0.42,0.60)	0.61 (0.50,0.76)	0.48 (0.40,0.59)
Age 50–69 years	0.89 (0.74,1.08)	1.06 (0.86,1.31)	1.17 (0.93,1.46)	1.48 (1.19,1.82)
≥ 70 years	0.67 (0.49,0.91)	1.18 (0.88,1.58)	0.77 (0.53,1.13)	1.75 (1.27,2.41)
Employment	1.02 (0.80,1.30)	1.33 (1.06,1.68)	0.80 (0.64,1.02)	0.92 (0.75,1.14)
Relative income	0.84 (0.77,0.90)	0.87 (0.80,0.95)	0.86 (0.78,0.94)	0.97 (0.89,1.06)
University education	1.06 (0.89,1.26)	1.05 (0.87,1.27)	1.22 (0.97,1.54)	1.05 (0.85,1.29)
Experience of epidemics	1.87 (1.53,2.27)	1.87 (1.51,2.33)	1.73 (1.37,2.17)	1.56 (1.28,1.90)
**Individual preparedness**				
Males	0.78 (0.66,0.92)	0.74 (0.62,0.89)	1.47 (1.19,1.80)	1.54 (1.29,1.84)
Age 50–69 years	1.45 (1.21,1.74)	1.40 (1.14,1.71)	1.16 (0.94,1.44)	0.83 (0.69,1.01)
≥ 70 years	1.49 (1.09,2.02)	1.26 (0.95,1.68)	1.44 (1.00,2.06)	0.83 (0.61,1.12)
Employment	1.07 (0.84,1.35)	0.86 (0.69,1.07)	1.05 (0.84,1.32)	0.99 (0.82,1.20)
Relative income	1.11 (1.03,1.20)	1.09 (1.00,1.19)	1.24 (1.13,1.37)	1.21 (1.12,1.31)
University education	1.08 (0.91,1.29)	1.13 (0.94,1.36)	1.31 (1.05,1.63)	1.02 (0.85,1.24)
Experience of epidemics	2.22 (1.82,2.70)	1.92 (1.55,2.38)	1.21 (0.98,1.50)	1.66 (1.39,1.98)
**Authority preparedness**				
Males	0.57 (0.48,0.67)	0.67 (0.56,0.79)	1.00 (0.82,1.23)	1.23 (1.03,1.46)
Age 50–69 years	1.16 (0.97,1.39)	1.35 (1.11,1.65)	1.21 (0.98,1.50)	0.97 (0.81,1.17)
≥ 70 years	1.22 (0.90,1.64)	1.07 (0.81,1.42)	1.38 (0.96,1.97)	1.18 (0.89,1.57)
Employment	1.20 (0.96,1.51)	0.82 (0.65,1.02)	0.82 (0.66,1.03)	1.05 (0.88,1.27)
Relative income	1.14 (1.06,1.23)	1.21 (1.11,1.31)	1.20 (1.10,1.32)	1.16 (1.07,1.25)
University education	1.09 (0.92,1.29)	1.12 (0.93,1.34)	1.02 (0.83,1.26)	0.98 (0.81,1.18)
Experience of epidemics	1.10 (0.91,1.33)	1.26 (1.02,1.54)	1.07 (0.86,1.32)	1.10 (0.92,1.30)
**Individual knowledge**				
Males	0.76 (0.65,0.90)	0.83 (0.70,0.99)	1.15 (0.94,1.41)	1.03 (0.87,1.23)
Age 50–69 years	1.12 (0.93,1.34)	1.14 (0.93,1.39)	1.23 (1.00,1.53)	0.87 (0.72,1.05)
≥ 70 years	1.27 (0.94,1.73)	0.94 (0.71,1.25)	0.92 (0.64,1.33)	0.73 (0.55,0.98)
Employment	0.92 (0.72,1.16)	0.97 (0.77,1.21)	1.25 (1.00,1.56)	0.89 (0.74,1.07)
Relative income	1.05 (0.98,1.14)	1.09 (1.01,1.19)	1.19 (1.08,1.31)	1.23 (1.14,1.33)
University education	1.75 (1.47,2.07)	1.57 (1.31,1.89)	1.00 (0.81,1.25)	1.34 (1.11,1.61)
Experience of epidemics	2.53 (2.07,3.07)	2.74 (2.21,3.40)	1.16 (0.94,1.44)	1.33 (1.12,1.58)
**Authority knowledge**				
Males	0.79 (0.67,0.92)	0.88 (0.74,1.05)	1.13 (0.92,1.38)	1.29 (1.08,1.53)
Age 50–69 years	1.01 (0.84,1.20)	1.14 (0.93,1.39)	1.43 (1.16,1.77)	0.92 (0.76,1.11)
≥ 70 years	1.06 (0.78,1.43)	0.66 (0.50,0.87)	1.64 (1.13,2.38)	0.89 (0.67,1.19)
Employment	1.04 (0.82,1.32)	0.79 (0.63,0.99)	0.97 (0.78,1.21)	0.93 (0.77,1.12)
Relative income	1.27 (1.18,1.38)	1.26 (1.16,1.37)	1.32 (1.20,1.45)	1.25 (1.16,1.35)
University education	1.47 (1.24,1.74)	1.20 (1.00,1.44)	0.96 (0.77,1.19)	0.98 (0.81,1.18)
Experience of epidemics	1.50 (1.24,1.81)	1.47 (1.19,1.80)	1.07 (0.86,1.33)	1.25 (1.05,1.49)

Direct experience of epidemics was the main driver of perceived likelihood of epidemics with a 2.49 to 3.16-fold higher odds. On the other hand, being male, older age, along with having a higher relative income were associated with a lower perceived likelihood of epidemics.

Focusing on the perceived impact of the epidemic at the individual and population level, direct experience of epidemics was related with a 1.22 to 1.52 higher odds, while being male and having a high relative income with a lower odds. Compared to younger generations, the elderly in Sweden perceived an important impact on the individual up to 3.5-fold increased odds in November. Concern among the elderly in Italy also increased between August and November for both individual (from 0.93 to 1.72 OR) and population impact (from 0.77 to 1.75 OR).

For perceived preparedness, having a higher relative income was positively associated with both individual and authorities’ preparedness, while the experience of epidemics was mainly related to a higher perceived individual preparedness (1.21 to 2.22 OR). Males compared to females perceived lower individual and authorities’ preparedness in Sweden, a relationship in the opposite direction was observed in Italy (see Table 2).

Finally, the experience of epidemics and university education were associated with perceiving a higher level of individual and authorities’ knowledge.

### How the excess mortality and national policies influence the risk perception

**Figure 3:**
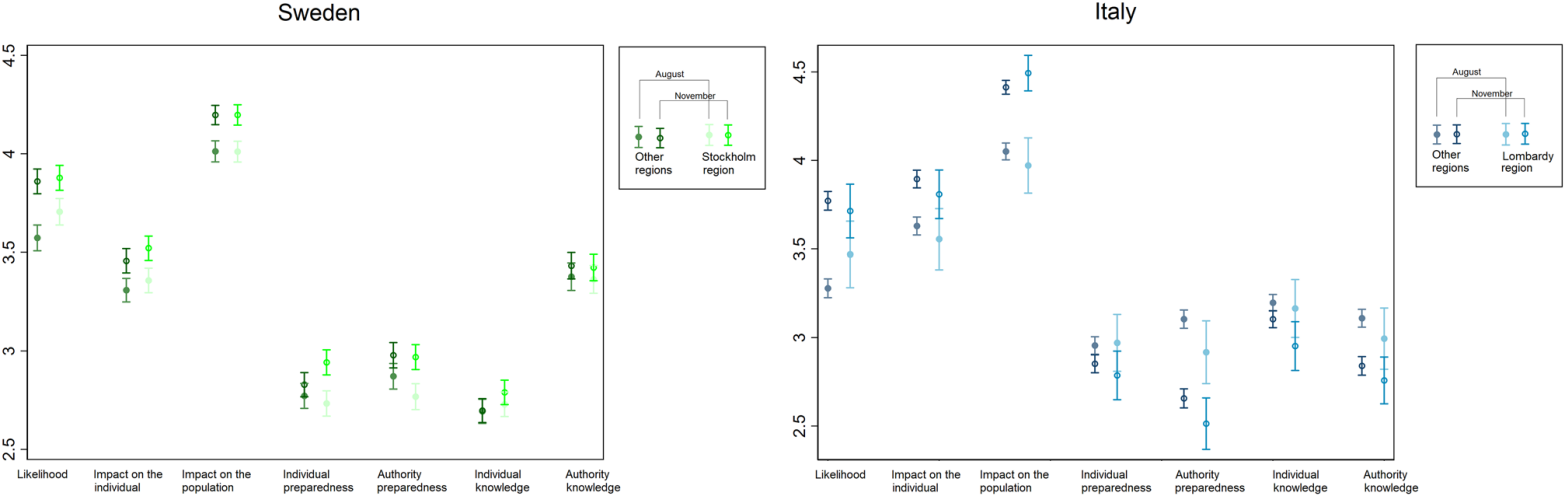
Means and 95% CIs for epidemic risk perception in Sweden and Italy by affected areas and period.

The first COVID-19 wave impacted Italy and Sweden to a different extent. Figure 3 compared the assessed risk perception in the most affected region, Stockholm region in Sweden and Lombardy region in Italy, with the rest of the country. Yet, the average risk perception did not reflect these geographical patterns and it was similar within countries. The only exception was a higher likelihood of epidemics in August, just after the first wave, in the most affected regions for both countries. Just at the beginning of the second wave (November), individuals living in the most affected regions perceived a lower individual knowledge in Italy and higher individual preparedness in Sweden compared to the rest of the population.

**Figure 4:**
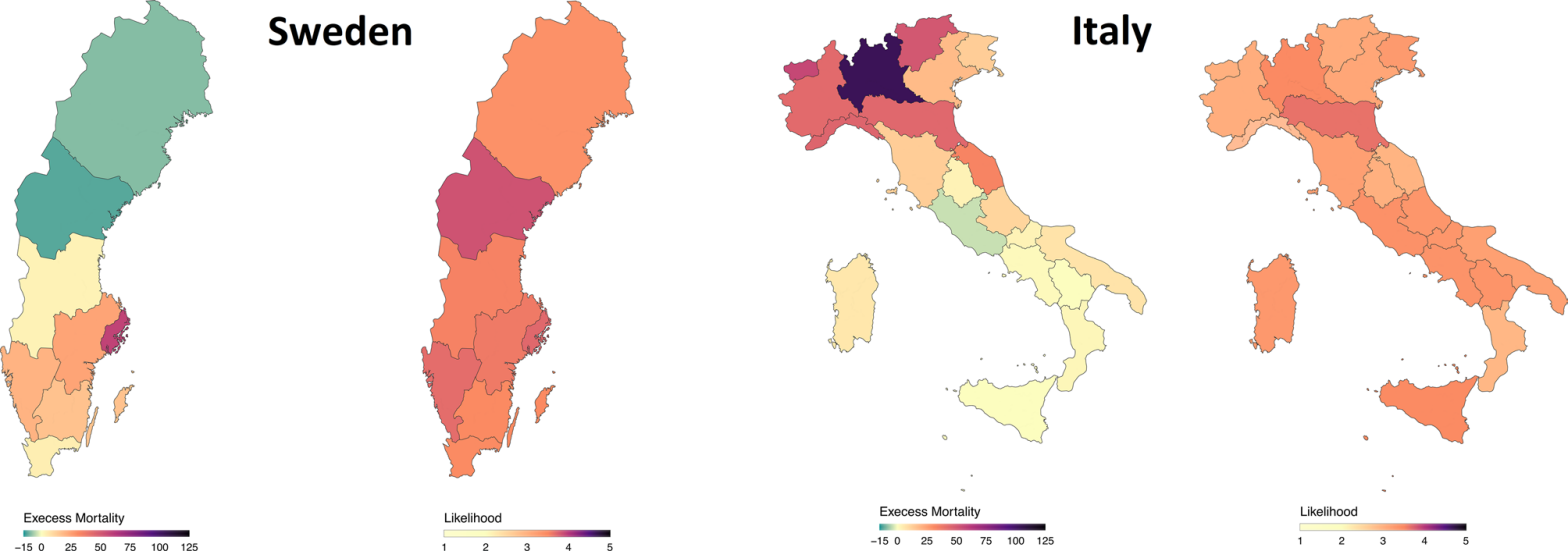
Perception of likelihood of epidemics after the first wave (August survey) and excess mortality during the first wave (15th February–15th May for Italy and 1st March–31st May for Sweden) stratified by region level. The figure was generate using R program and Inkscape (author: E.M.).

Figure 4 shows the excess mortality and likelihood of epidemics at regional level in Italy and Sweden. Despite significant differences in excess mortality among Italian and Swedish regions, the perception of likelihood of epidemics was quite similar within countries. Looking at the role of excess mortality as a predictor of risk perception domains, the excess mortality seemed only to marginally explain the likelihood of epidemics while no association was found with other domains (Table 3).

**Table 3:** Association between excess mortality (for each 10% increase) and domains of risk perceptions.

	Sweden ORs (95% CIs)	Italy ORs (95% CIs)
Likelihood	1.07 (1.03,1.11)	1.03 (1.01,1.06)
Impact on the individual	1.02 (0.98,1.06)	0.99 (0.97,1.01)
Impact on the population	1.01 (0.97,1.04)	0.98 (0.96,1.00)
Individual preparedness	0.97 (0.93,1.00)	1.00 (0.98,1.02)
Authority preparedness	0.94 (0.91,0.97)	0.97 (0.95,0.99)
Individual knowledge	1.01 (0.98,1.05)	0.98 (0.96,1.00)
Authority knowledge	0.95 (0.92,0.98)	0.97 (0.95,0.99)

Models adjusted for gender, age and relative income.

Moving to the role of national policy responses, Fig. 1 describes the change over time of the level of measures (Stringency Index, ranging from 0 to 100, 100 = strictest) implemented in Italy and Sweden^18^. The level of restrictions was higher in Italy (maximum 93.4) than in Sweden (maximum 64.8) during the first wave. This index was positively associated with perceived likelihood, impact along with individual knowledge and preparedness and negatively associated with authority knowledge and preparedness (Table 4).

**Table 4:** Association between the level of national policy response (Stringency index) and domains of risk perceptions.

	ORs (95% CIs)
Likelihood	1.74 (1.52,2.00)
Impact on the individual	3.06 (2.65,3.53)
Impact on the population	3.28 (2.84,3.78)
Individual preparedness	1.22 (1.06,1.40)
Authority preparedness	0.72 (0.63,0.82)
Individual knowledge	1.80 (1.57,2.06)
Authority knowledge	0.45 (0.39,0.51)

Models adjusted for gender, age and relative income.

## Discussion

In both countries, there was an overall intensification in average risk perception from August to November 2020, characterized by an increase in perceived likelihood and impact of epidemics, along with a decrease in perceived individual and authorities’ preparedness and knowledge. This may be of concern as, according to the Protection Motivation Theory^19,20^, a high threat appraisal paired with a low coping appraisal may result in the choice of not protecting oneself. In the case of COVID-19, this poses a risk not only to the individual, but also to others around them. Being male, older age along with having a higher relative income were associated with a lower perception of the likelihood of epidemics, while excess mortality was marginally related to a higher odds. People in Italy had a higher increase in perception of likelihood and impact of epidemics compared to people in Sweden and a greater decrease in perceived preparedness and knowledge were observed over time. Regional differences in excess mortality within each country only marginally explained differences in the risk perception.

How a pandemic affects a population stems from the interplay of different factors: population characteristics, prompt response to the spreading, and preparedness of the health care system^12^. While preparedness of the health care system requires years to establish and is continuously evolving, a prepared contingency plan can be implemented within days. From past pandemics, we know that the success of policies and responses to reduce the infection rate and to avoid epidemics depends on risk perception and behavioral adaptation to such policies and response measures^21^. Increasing COVID-19 cases, media coverage and implementation of more restrictive measures may have contributed to the increase in the average risk perception from August 2020 to the beginning of the second wave of the COVID-19 pandemic (November 2020) in our sample. Cross-sectional surveys on risk perception of the COVID-19 pandemic found a positive association between higher risk perception and higher compliance to preventive measures^22,23^. Thus, the increased risk perception in the present study may result in people applying behavioral measures such as social distancing, hand washing, avoidance of public places and transports, and wearing face masks.

As reported in previous studies, our findings support the theory of lower risk perception among individuals with a traditionally higher status in society^24^. Men, the elderly population and individuals with a higher income perceived a lower risk compared to women, younger generations and individuals with lower income, respectively as shown in other surveys^25,26^. Media coverage and public messages from the authorities can increase awareness of the impact of COVID-19 infection, in turn facilitating engagement in protective behaviors, especially among men and the elderly—two groups at risk for severe consequences if infected^27^. Along with this, governments should consider the negative consequences that disadvantaged socio-economic groups are facing and should address rising inequalities following the pandemic.

How excess mortality and national policies during the first wave affect the epidemic risk perception is another important aspect to disentangle. In the present study, excess mortality at regional level was marginally associated with perceived likelihood of epidemics. Comparing Italian and Swedish authorities’ responses during the COVID-19 pandemic may help to untangle the role of national policies. The more stringent measures in Italy during the first wave may explain the higher risk perception in August, and the application of stronger measures at the beginning of the second wave (November 2020) may justify a higher increase of risk perception in Italy than in Sweden over three months. Generally, national policies rather than excess mortality may influence the risk perception since national recommendations and restrictions affect all the population, while few households are affected by the epidemic, even in a scenario with 100% excess mortality.

Findings from this study shed light on the change of public risk perception during the current pandemic informing the scientific community and policymakers. At odds with other threats, individuals have a double role during a pandemic: protecting themselves and safeguarding vulnerable groups at the greatest risk. Implementing personal hygiene measures and adhering to national policies are both required to slow the speed of infection and reduce the burden on the health care system. In our study, high levels of risk perception resulted in a lower trust in authorities with a possible shift of responsibility from trusted authority to individuals. This reduction in perceived authority knowledge and preparedness was stronger in Italy, a country where the restrictions were imposed, compared to Sweden, where the policies have been mainly based on recommendations. A reduction over time of trust in the authorities is of concern since it may affect the adherence to imposed measures. Modern democracies rely on a social contract where each individual should contribute to the collective well-being in proportion to their abilities and exercise their right to vote through their representatives^28^. Imposing measures to limit personal freedom has a positive effect in the short term to reduce the spread of infection. From a long-term perspective, a paternalistic approach may lead to the emergence of conflicts, non-adherence of imposed measures, and the disruption of the social contract. Policymakers should encourage community involvement and make communities the centre of the response to this pandemic. This approach can help to obtain adherence to the necessary preventive measures and defend the social contract on which modern democracies are based.

Along with this, globalization brings new challenges on how to communicate risk in public health and may hamper the efforts of community engagement. Social media algorithms, so called filter bubbles, contribute to create like-minded digital communities and facilitate the spreading of conspiracy theories. The vaccination hesitancy movement during the vaccination campaign against COVID-19 infection is emblematic of such a phenomenon. The spreading of false information on possible vaccine side effects has influenced the willingness to be vaccinated and has induced governments to impose measures to guarantee public and individual health limiting the freedom of the unvaccinated population^29,30^.

A number of additional limitations should be kept in mind when interpreting findings from our study. First, domains of risk perceptions should be interpreted in terms of change over time rather than absolute values given that cultural belief and other country-level factors may affect absolute values. Second, we considered independent samples of the population in the first and second survey wave. Although the samples should be considered representative of the general populations for age and gender, other individual differences may influence the changes over time. Third, this study does not allow to disentangle the specific role of restrictive measures and media coverage as determinants of risk perception. Finally, while the proportion of missing data for variables was low and is unlikely to have impacted the final estimates, we cannot exclude a selection of participants due to a low initial participation rate that may influence the reported risk perception. However, the strategy applied for selecting the sample should have minimized this source of bias.

Conclusively, our findings indicate an increased average epidemic risk perception during the COVID-19 pandemic from August to November 2020. Being male, elderly and a high income was related with a lower risk perception. Considering country-level factors, national policy response rather than excess mortality was an important determinant of the risk perception. In the future months, efforts should be directed towards monitoring the change of risk perception over time, investigating if a higher risk perception is associated with adherence to national recommendations also for the COVID-19 pandemic and untangling the role of media coverage and recommendations on risk perception.

## Methods

### Study population

We included data from an anonymous survey on public risk perception carried out in Italy and Sweden in two different periods of the COVID-19 pandemic. Detailed information on the study has been published elsewhere^31^. In brief, the survey explores the public risk perception for nine threats (epidemics, floods, droughts, earthquakes, wildfires, terror attacks, domestic violence, economic crises, and climate change). Data were collected throughout a one-week period in August and November 2020. The samples were independent, and derived from two existing survey panels of 100,000 individuals in each country, set up by Kantar Sifo marketing research company^32^, and should be considered representative of the Swedish and Italian population for sex and age. Around 8000 individuals in the pool were invited to participate, if they did not reply, up to two reminders were sent. The capital regions were overrepresented: with a 1/9 sampling ratio in Italy and 4/6 sampling ratio in Sweden) (Supplementary Fig. 1). The missing data was quite low, < 5% for almost all variables included in the study, with the exception of political orientation in the Italian context where 20% of individuals preferred not to answer. The total sample included 8322 individuals. 4154 individuals participated in the survey in August (N = 2033, mean age 50.3 years, 53.0% of females in Italy and N = 2121, mean age 49.3 years, 49.9% of females in Sweden) and 4168 in November (N = 2004, mean age 49.4 years, 50.7% of females in Italy and N = 2164, mean age 47.9 years, 51.4% of females in Sweden).

Individuals that lived in the capital region were overrepresented, specific weights were applied in the analysis to take this into account. The present study was approved by the Italian Research Ethics and Bioethics Committee (Dnr 0043071/2019) and the Swedish Ethical Review Authority (Dnr 2019-03242). The study was carried out in accordance with the ethical standards set by the European Union under Horizon 2020 (EU General Data Protection Regulation and FAIR Data Management). Participants were informed that the participation was voluntary and they give their inform consent to participate in this study when completing the survey.

### Risk perception of epidemics

The present study considered the public risk perception of epidemics considering seven domains: the likelihood of epidemics, epidemic impact on the individual and on the population, individual and authority preparedness, individual and authority knowledge of epidemics with a Likert-type scale ranging from 1, minimum to 5, maximum.

### Predictors of the risk perception

Information on direct experience of an epidemic and socio-economic factors such as age, gender, employment (yes vs. no), relative income (from 1 to 5), university education (yes vs. no) were collected in the survey and included in the present study as possible predictors of risk perception.

### Excess mortality

Excess mortality at regional level in Italy and Sweden during the first wave of the COVID-19 pandemic (15th February–15th May for Italy and 1st March–31st May for Sweden) was considered in the study. Regional level was defined according to Nomenclature of Territorial Units for Statistics (NUTS) 2 classification of the European Union^33^. We retrieved data on excess mortality among the Italian regions from Scortichini et al.^17^. To estimate the excess mortality in Sweden, we compared the COVID-19 outbreak with the pre-outbreak period. A two-stage interrupted time-series approach, that relied on a Poisson model with a function that constrains the excess risk as null at the beginning of March 2020, was used to calculate the excess mortality at the Swedish regional level^34^. The model was adjusted for time-varying confounders such as (i) seasonality using a natural spline term with 3 knots, (ii) indicators for the day of the week, (iii) air temperature using a term for the mean daily temperature. The information on temperature was retrieved from the ERA-5 reanalysis data set on the Copernicus climate data store^35^. We performed mixed-effects Poisson regression models with a random term for NUTS3 administrative units to calculate the excess mortality at regional (NUTS2) level taking into account the heterogeneity among NUTS3 administrative units.

### National policy response

The Stringency index^18^ is a national response index and is used to quantify the measures implemented in response to the COVID-19 pandemic. The Stringency Index is a daily measure at country level that considers nine domains: school closures; workplace closures; cancellation of public events; restrictions on public gatherings; closures of public transport; stay-at-home requirements; public information campaigns; restrictions on internal movements; and international travel controls. In this paper, the level of national policy response was used as an ecological variable with four levels (Sweden up to August, Italy up to August, Sweden up to November and Italy up to November) and was defined as the area under the curve of the Stringency Index for each country, between two successive days up to the 5th August 2020 (first survey) and the 4th November 2020 (second survey). This measure was standardized on the value of Sweden in August (considered as the reference).

### Statistical analysis

Possible differences in means and confidence intervals for seven items of risk perception between countries and over time were graphically presented using forest plots and stratified by country and period. Effect modification by country and period was examined using ordinal logistic regression models with risk perception (independent variables) and country and period as dependent variables. Results were presented as (i) Odds Ratios (ORs) for each country and period strata, (ii) ORs for country within strata of period and for period within strata of country, and (iii) interaction measures on additive and multiplicative scales^36^.

Second, multivariable ordinal logistic regression models were performed to evaluate the association of gender, age, employment, relative income, university education and experience of epidemics as possible predictors with the seven domains of risk perception (independent variables). The analysis was stratified by country and period.

Third, we examined if the risk perception varied according to which extent an area was affected by the first wave of the COVID-19 pandemic. We compared the means and confidence intervals for seven items of risk perception between the most affected region in terms of excess mortality (Stockholm region in Sweden about 60% excess mortality and Lombardy region in Italy about 100% excess mortality) and comparison with the rest of the country. Then, ordinal logistic regression models were performed to examine if the excess mortality at regional level (dependent variable) was associated with domains of risk perception (independent variables) stratifying for country and adjusting for gender, age, and relative income. Finally, the association between the level of implemented measures and risk perception was explored using adjusted ordinal logistic regression models.

The use of ordinal logistic regression models relied on the assumptions that the effect was linear on the log scale and that each independent variable had an identical effect for one unit increase of the ordinal dependent variable (proportional odds). Along with this, the goodness of fit of the ordinal logistic models was tested using the Deviance goodness of fit test. No multicollinearity among independent variables and correlation among errors from the models were detected.

As has been suggested in the literature, there are considerable risks in misinterpreting p-values^37^. Therefore, we opted to interpret the estimates in terms of possible direction of the effects and using ORs and 95% Confidence Intervals (CIs), which contain information on significance. Specifically, the width of the confidence interval and the size of the p-value are related: the narrower the interval is, the smaller the p-value is. Moreover, the confidence interval gives additional information that is related to the magnitude of the effect being investigated.

Statistical analyses were performed using Stata version 15.0 (StataCorp, College Station, TX, USA) and R version 4.0.3^38^.

## Supplementary Information

Supplementary Information.

## Data Availability

The datasets generated and analysed during the current study are available in the Zenodo repository titled “A comparative dataset on public perceptions of multiple risks during the COVID-19 pandemic in Italy and Sweden”, https://zenodo.org/record/5653322#.YoIf4OhBw2x. Code for processing and graphically representing the data is available at https://github.com/eleraf/epidemics-risk-perception.
