# Supplementary material for: Epidemic risk perceptions in Italy and Sweden driven by authority responses to COVID-19
Source: Sci Rep. 2022 Jun 3;12:9291. doi: 10.1038/s41598-022-13218-w (PMC9164564; doi:10.1038/s41598-022-13218-w)
Supplement: Supplementary file 1 — Supplementary Information. [file 41598_2022_13218_MOESM1_ESM.docx]

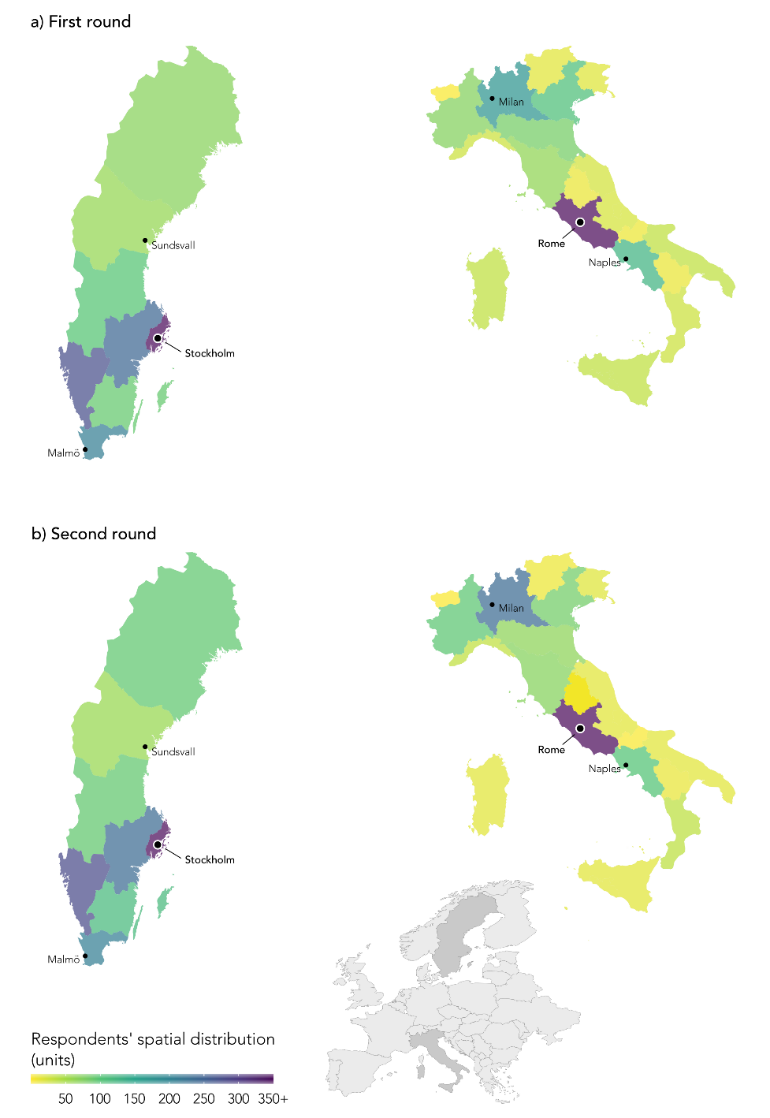


Figure S1: Geographical distribution of the participants. The figure was generated using R version 4.0.3 (ggplot2 library)
